# Evolution of cubic membranes as antioxidant defence system

**DOI:** 10.1098/rsfs.2015.0012

**Published:** 2015-08-06

**Authors:** Yuru Deng, Zakaria A. Almsherqi

**Affiliations:** 1 Institute of Biomedical Engineering and Health Sciences, Changzhou University, Changzhou, Jiangsu 213164, People's Republic of China; 2 Department of Physiology, Yong Loo Lin School of Medicine, National University of Singapore, Singapore 117597, Republic of Singapore

**Keywords:** cubic membranes, oxidative stress, plasmalogens, very long-chain polyunsaturated fatty acids, structural antioxidant, RNA protection

## Abstract

Possibly the best-characterized cubic membrane transition has been observed in the mitochondrial inner membranes of free-living giant amoeba (*Chaos carolinense*). In this ancient organism, the cells are able to survive in extreme environments such as lack of food, thermal and osmolarity fluctuations and high levels of reactive oxygen species. Their mitochondrial inner membranes undergo rapid changes in three-dimensional organization upon food depletion, providing a valuable model to study this subcellular adaptation. Our data show that cubic membrane is enriched with unique ether phospholipids, plasmalogens carrying very long-chain polyunsaturated fatty acids. Here, we propose that these phospholipids may not only facilitate cubic membrane formation but may also provide a protective shelter to RNA. The potential interaction of cubic membrane with RNA may reduce the amount of RNA oxidation and promote more efficient protein translation. Thus, recognizing the role of cubic membranes in RNA antioxidant systems might help us to understand the adaptive mechanisms that have evolved over time in eukaryotes.

Biomembranes are traditionally viewed as flat sheets of phospholipid bilayers dividing the cytoplasm into multiple subcellular compartments with specialized functions. However, biomembranes may also fold up into three-dimensional periodic arrangements termed ‘cubic membranes’ (figure 1) [1,2]. Cubic membranes can be observed in virtually any membrane-bound subcellular organelles [3]. Such induced membrane transition changes are frequently accompanied by alterations in cellular oxidative stress responses, such in neoplasia, inflammation and viral infection conditions [4,5]. We have suggested on the basis of these observations that cubic membrane formation may be associated with oxidative stress [6]. In living organisms, antioxidant enzymes form the first line of defence against reactive oxygen species (ROS) in the cellular environments [7]. These enzymes work in tandem to decrease the damaging effects of ROS in the cells.

**Figure 1.**
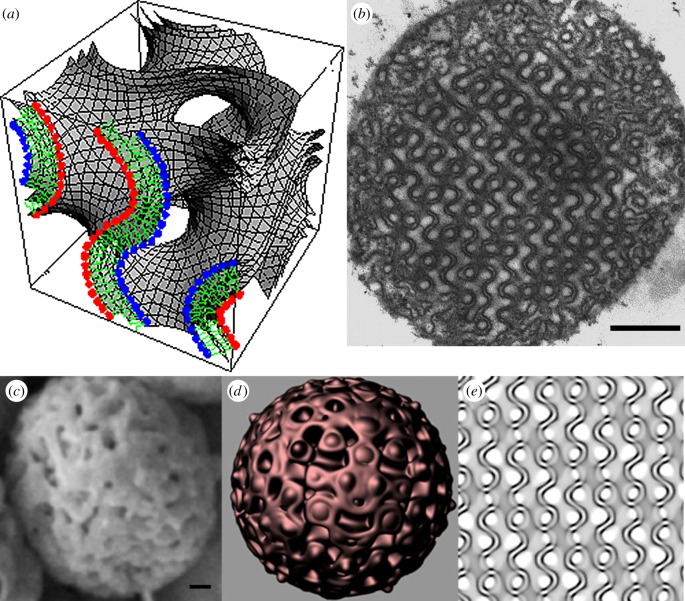
Cubic membrane architecture. (*a*) Three-dimensional mathematical model representing the phospholipid bilayer of cubic membrane organization. (*b*) Two-dimensional transmission electron micrograph of the same three-dimensional model presented in (*a*). (*c*) Scanning electron micrograph and its corresponding (*d*) three-dimensional and (*e*) two-dimensional computer simulation model of cubic membranes found in the mitochondria of 10-day starved amoeba *Chaos* cells. Scale bars, (*b*) 500 nm and (*c*) 100 nm.

However, living organisms developed several other defence mechanisms to cope with oxidative stress. For example, in the unicellular organism *Escherichia coli*, levels of fumarase C, which is insensitive to superoxide anions, increase during oxidative stress, probably to replace fumarases A and B which are susceptible to damage by superoxide anions [8]. In other organisms, ‘sacrificial agents' are oxidized preferentially in oxidative stress conditions to protect important biomolecules [9]. These observations suggest that living organisms may use a wide range of biomolecules and mechanisms other than antioxidant enzymes to ameliorate the damaging effects of ROS.

It had been established that starved amoebae (*Chaos carolinense*) contain greater levels of free radicals than fed amoebae [6]. Starvation induces cubic membrane formation in amoeba *Chaos* mitochondria [10]. Similarly, in the higher plants, ‘light starvation’ (absence of the light) also induces cubic membrane formation in the photosynthetic thylakoid membranes, prolamellar bodies [11]. The transformed inner mitochondrial membranes into cubic organization in the starved amoeba *Chaos* exhibit a high content of very long-chain polyunsaturated fatty acids (VLC-PUFAs), specifically the C22 : 5n-6 modified phosphatidylcholine plasmalogen, phosphatidylethanolamine plasmalogen and phosphatidylinositol species, that appear to be critical for development and maintaining highly ordered yet curved interwoven cubic membrane structures [12].

We note that the mitochondria with cubic membrane organization isolated from starved amoeba *Chaos* interact sufficiently with short segments of phosphorothioate oligonucleotides (PS-ODNs, resemble RNA in biological systems). We also study the ability to provide ODN uptake via cubic membranes [13]. Specifically, we have observed ODNs condensed within the convoluted channels (most likely within the mitochondrial intermembrane space rather than the matrix) of cubic membranes by an unknown passive targeting mechanism [13]. Moreover, the interaction between ODNs and cubic membranes is sufficient to retard ODN oxidation by free radicals *in vitro* (figure 2). Hence, the close similarity between the ODNs used experimentally and the RNAs. Cubic membranes, therefore, may act as a ‘protective’ shelter minimizing or preventing the oxidation of biologically essential macromolecules such as RNAs.

**Figure 2.**
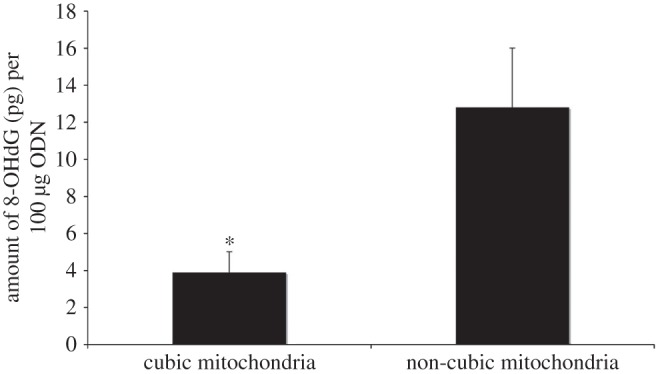
Bar graph depicts the difference in the amount of 8-OHdG (pg) per 100 μg of ODN in a mixture containing cubic mitochondria and that containing non-cubic mitochondria. The mixture containing non-cubic mitochondria has approximately four times as much 8-OHdG as that containing cubic mitochondria. In this experiment, mitochondria with cubic membrane organization were isolated from 7-day starved amoeba *Chaos* and the mitochondria without cubic membrane organization were isolated from mouse liver. Same amount of mitochondria protein was incubated with the same amount of ODN in separate tubes before the mixture was exposed to superoxide anions generated by the Fenton reaction. After exposure to the Fenton reaction, ODNs were isolated and assessed for oxidative damage. *p-value < 0.05.

It is believed that the alterations of gene expression and faithful translation of RNAs are the key factors of the metabolic changes essential for cell survival and the rapid organismal adaptation to new environmental stimuli. Oxidative damage to both coding and non-coding RNAs may therefore affect the regulation of gene expression and, potentially, result in the failure of protein synthesis. This failure may impair the organismal capacity of flexibly adapting to a novel or unusual internal or external environmental stimulus [14]. Furthermore, experimental data suggest that oxidative damage of RNAs may be involved in the cellular patho-mechanisms of several diseases and cell survivability [14]. In a eukaryotic cell, nearly all the DNA in the cell is sequestered within the nucleus. The nuclear envelope delimits the compartment and physically limits the interaction between the nucleus and the cytoplasm. Thus, RNAs rather than DNAs are ‘vulnerable’ targets of oxidation because of their biochemical structure, relatively abundant in the cell and they are mostly located in the vicinity of ROS producing organelles such as mitochondria and peroxisomes [15–21]. As a consequence, oxidative damage to RNA rather than DNA may be the more proximate cause of impairment in cellular performance and adaptability. Specific protective mechanisms of RNAs and the control of their degradation would be therefore expected to be present in cells [22].

Plasmalogens are a unique class of ether phospholipids with a vinyl ether bond at *sn*-1 position and enriched in PUFAs at *sn*-2 position of the glycerol backbone [23]. They form the major components of cubic membrane phospholipids and play a critical role in membrane plasticity and transformation into cubic structure [12]. We are interested whether the unusually high amounts of VLC-PUFAs and plasmalogens in cubic membranes and their ability to interact with ODNs play a role in the defence system of RNAs and, therefore, of the gene expression regulatory system in living organisms. Although the functions of VLC-PUFAs and plasmalogens in cubic membranes are still far from being fully understood, recent studies have shown that VLC-PUFAs may affect the expression of many genes and that these effects appear to be independent of any changes in membrane composition [16,24,25].

A number of studies have shown that plasmalogens are protective in lipid peroxidation [26,27]. Sindelar *et al.* [27] demonstrated that brain phospholipids with and without plasmalogens in separate liposomal systems were subjected to oxidative stress. The results revealed that in the presence of plasmalogens, markers for lipid peroxidation were significantly decreased. This implies that plasmalogens protect PUFAs from damage. Although the mechanism for this phenomenon has not been elucidated, it may involve vinyl ether bonds in plasmalogens which are more susceptible to oxidative attack than via ester bonds in phospholipids [28].

The free radical species formed during the peroxidation of the vinyl ether bond may either be more stable or be less efficient to abstract hydrogen than the alkyl radicals produced during the peroxidation of PUFAs [27]. Also, it is likely that the oxygenated vinyl ether radicals are broken down into water-soluble radical compounds which are unable to further propagate the oxidation cascade [27].

In addition to the mitochondrial antioxidant enzymes, plasmalogens can limit the diffusion of ROS within the different compartments of cubic membrane because of their proneness to be peroxidized. This mechanism would limit the intercellular transmission of ROS and that the oxidative cascade might spread to RNAs segregated into the inner compartments of cubic membranes. Some support for this proposition comes from experimental observations where cubic membranes strikingly correlate with viral infections; notably, RNA viruses [5]. Viral entry, proliferation and release are processes closely linked to cubic membrane formation [5]. Generating cubic membrane during viral genome proliferation may provide a protective membrane environment to protect the viral RNA from oxidative damage and facilitate faithful genomic transcription and translation.

Plasmalogen oxidation and VLC-PUFA peroxidation in phospholipids of cubic membranes have a number of negative downstream effects on the physical properties and structure of the membranes, such as the decrease in their fluidity and increases in lamellar membrane formation rather than cubic organization. However, an increasing body of evidence suggests that peroxidized phospholipids can be repaired by the enzymes phospholipase A2 and acyl-transferase [29]. While unesterified VLC-PUFAs present in the cytoplasm can be incorporated into the cellular membranes to replace the oxidized molecules.

In view of these observations, we propose here an integrative model (figure 3) where natural selection has operated in order to optimize a molecular composition to have a three-dimensional membrane shape that is enriched in plasmalogens containing VLC-PUFAs. Plasmalogens that carry VLC-PUFAs would facilitate cellular membrane transformation from the lamellar into the cubic arrangement. In this model, cubic membranes interact with short segments of ODNs such as RNAs to segregate RNAs and possibly other translationary systems into the inner compartments of cubic membranes. The high susceptibility of VLC-PUFAs and plasmalogens in cubic membranes to oxidation further retards RNAs oxidation. Cubic membranes may indirectly play a role in the defence system of RNAs. Our proposal implies biochemical pathways and highly ordered three-dimensional membranes in shape that are functionally integrated, possibly resulting in an evolutionary module such as cubic membranes for cell survival. The understanding of the biological relevance of RNA protection by cubic membranes and of its evolutionary underpinnings may explain fundamental aspects of selective pressures modulating the awareness of evolution in eukaryotic cells.

**Figure 3.**
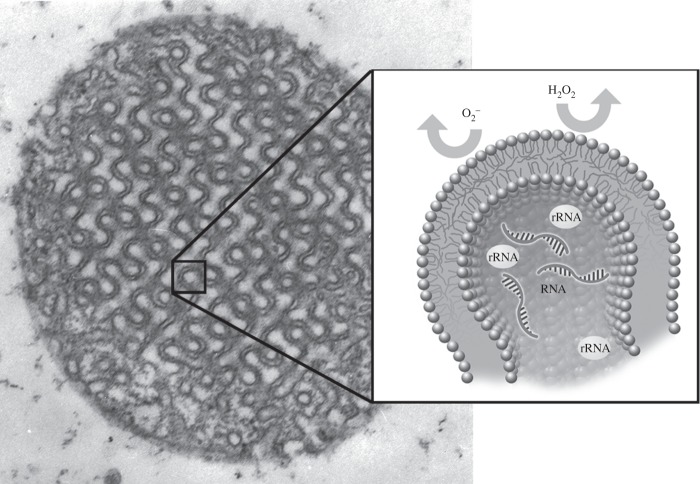
The proposed mechanism of antioxidant defence system of cubic membranes in oxidative stress conditions. The high content of plasmalogens in cubic membranes may preferentially interact with superoxide anions protecting the biomolecules in cubic membrane channels. This may provide a safe environment for RNA and other protein synthesis machinery molecules (e.g. ribosomal RNA) within the internal compartments of the cubic membranes.
